# Experiences with long acting injectable ART: A qualitative study among PLHIV participating in a Phase II study of cabotegravir + rilpivirine (LATTE-2) in the United States and Spain

**DOI:** 10.1371/journal.pone.0190487

**Published:** 2018-01-05

**Authors:** Deanna Kerrigan, Andrea Mantsios, Miguel Gorgolas, Maria-Luisa Montes, Federico Pulido, Cynthia Brinson, Jerome deVente, Gary J. Richmond, S. Wilson Beckham, Paige Hammond, David Margolis, Miranda Murray

**Affiliations:** 1 Department of Health, Behavior & Society, Johns Hopkins University Bloomberg School of Public Health, Baltimore, Maryland, United States of America; 2 Hospital Fundación Jiménez Díaz, Madrid, Spain; 3 Hospital La Paz, Madrid, Spain; 4 Hospital Universitario Doce de Octubre, i+12, Madrid, Spain; 5 Central Texas Clinical Research Corporation, Austin, Texas, United States of America; 6 Living Hope Foundation, Long Beach, California, United States of America; 7 Independent Researcher, Fort Lauderdale, Florida, United States of America; 8 ViiV Healthcare, Raleigh-Durham, North Carolina, United States of America; 9 ViiV Healthcare, London, United Kingdom; University of Washington, UNITED STATES

## Abstract

Challenges with adherence to daily oral antiretroviral therapy (ART) among people living with HIV (PLHIV) have stimulated development of injectable long-acting (LA) regimens. We conducted 39 in-depth interviews with participants and providers in a Phase IIb study (LATTE-2) evaluating an injectable LA regimen in the U.S. and Spain. Interviews exploring participant and provider attitudes and experiences with LA versus oral ART were audiotaped, transcribed and analyzed using thematic content analysis. Participants described the convenience of LA injections versus daily pills and emotional benefits such as minimized potential for HIV disclosure and eliminating the “daily reminder of living with HIV.” Providers recognized benefits but cautioned that LA candidates still need to adhere to clinic visits for injections and raised questions around ongoing clinical management. LA was seen as preferable to daily oral ART among PLHIV. Further research is needed regarding appropriate candidates, including with women and “non-adherent” populations across settings.

## Introduction

Three decades after the emergence of HIV on the international stage, the epidemic continues with an estimated 36.7 million people living with HIV (PLHIV) globally [1]. In 2015, there were 2.1 million new HIV infections and 1.1 million AIDS-related deaths [2]. Critical improvements in addressing the epidemic have occurred over the years, including a 38% decline in HIV incidence since 2001 and a 35% reduction in AIDS mortality since 2005 [3].

Access to antiretroviral therapy (ART) to treat HIV has led to dramatic reductions in AIDS-related morbidity and mortality and improvements in the health and wellbeing of PLHIV [4]. Earlier use of ART or what is now known as treatment as prevention (TasP) has been shown to prevent the transmission of HIV by reducing viral load of PLHIV, thereby decreasing their infectiousness [5–8]. TasP was first evaluated in the landmark HPTN-052 trial conducted among serodiscordant couples [9, 10], where early initiation of ART delayed time to AIDS events for HIV-infected participants and resulted in a 96% reduction in the risk of HIV transmission to a serodiscordant partner [9, 11].

Evidence of the real-world effectiveness of TasP is more limited. A large prospective cohort study in South Africa found that reductions in community viral load due to increased ART coverage were associated with a significant decline in individual HIV acquisition [12]. Similar results have been observed in San Francisco [13] and British Columbia [14]. However, numerous barriers to large-scale implementation of TasP exist both in terms of health systems and individual behavior. In particular, TasP not only requires infected individuals to be diagnosed, linked to care, and started on ART, but they must also adhere to daily oral ART indefinitely.

The existing literature has highlighted sub-optimal adherence to ART across settings and populations [15]. A global meta-analysis estimated that 55% of PLHIV taking ART in North America and 77% in Africa have suboptimal adherence [16]. Barriers to daily oral ART adherence range from those imposed by characteristics of pill regimens and their side effects, health care systems and provider communication issues [17–19], to psychosocial and structural barriers related to the social context surrounding HIV and ART. These can include substance use and mental health [20–23], inequitable gender norms and roles [21, 24], as well as stigma and discrimination associated with HIV and marginalized populations disproportionately affected by the epidemic [25, 26].

Injectable long-acting (LA) ART may address some of these barriers to adherence, as it would be delivered to patients in the form of monthly or bi-monthly injections in a medical setting, as opposed to a daily pill. Two antiretroviral drugs, cabotegravir (CAB) and rilpivirine (RPV), are currently being studied for use as injectable LA ART with a Phase II trial having now completed primary analyses and Phase III trials currently underway. To date, studies have found these regimens to be effective and to have acceptable tolerability and safety profiles, via quantitative assessments of patient experiences as part of trial conduct [27–29]. CAB and RPVhave also exhibited potent antiviral activity and health outcomes, such as ability to maintain levels of viral suppression, following initial oral induction with combination ART as subjects continuing on a combination daily oral regimen [30]. Here we qualitatively explore the views and experiences of PLHIV and their providers participating in the LATTE-2 trial in the United States and Spain to inform future research and potential programmatic roll-out of LA ART across settings.

## Methods

### Study design

We undertook a cross-sectional qualitative study of participants and providers from the LATTE-2 trial, a phase IIb study accessing the safety, tolerability, and acceptability of LA CAB and RPV for the treatment of HIV. The trial included 309 treatment naïve HIV-infected participants. All participants were initially provided a three-drug (cabotegravir, abacavir, & lamivudine) oral induction regimen. Those who achieved viral suppression during the induction period were randomized to receive (1) LA injections every 4 weeks, (2) LA injections every 8 weeks, or (3) continue on the daily oral regimen [31].

### Data collection procedures

Twenty-seven trial participants, from the LA 4 or 8 week arms, and 12 providers were recruited from LATTE-2 sites in Austin, TX; Long Beach, CA; Ft Lauderdale, FL; and three clinics in Madrid, Spain. The six clinical sites were selected based on geographic diversity and the number of people enrolled in the trial in those settings. Ten to fifteen individuals is generally understood as a sufficient sample to begin to describe a phenomenon of interest from the view of a given population group within the field of qualitative research [32]. We thus included 10–15 patient participants in each of the two settings. The three U.S. study sites were generally smaller clinical settings than the three sites in Madrid with one of the U.S. sites seeing an average of 55 patients per month compared to one of the Madrid sites, which operated within a large public hospital, seeing an average of 650 patients per month. Reflecting this difference in clinic size, the U.S. sites also had smaller numbers of participants enrolled in LATTE-2 than the sites in Madrid. Data collection occurred between November 2015 and January 2016. LATTE-2 staff contacted trial participants (either in person or by phone) and provided information on the qualitative study and its objectives. Potential participants were then referred to the study team for an in-person meeting where informed consent and interviews were conducted onsite at the clinics. The LATTE-2 trial was primarily male (92%) limiting the ability of the study staff to recruit more than a few female participants. To the extent possible, we sought diversity in terms of race/ethnicity and sexual orientation. The LATTE-2 staff also assisted the study team to recruit individuals with varying levels of satisfaction with study participation and those that had and did not have adverse effects.

Patient interviews were held at the local LATTE-2 clinic sites in private rooms. All interviews were conducted by two bilingual study researchers trained in qualitative methods and research ethics. The interviews were conducted in the relevant local language (English in the U.S. and Spanish in Spain) and were audiotaped. In an effort to ensure language used was appropriate for the Spanish setting versus Central or Latin America, professional Spanish using neutral words for which meaning does not vary across regions was used. Semi-structured interviews were facilitated by a flexible guide of open-ended question to explore participant views and experiences related to LA ART. Topics of discussion included injection experiences, perceived advantages and disadvantages of daily oral and LA injectable ART, perceived appropriate candidates for LA ART, and future service delivery preferences. Participants were compensated $50 for the one-time interview. The study was approved by the Institutional Review Boards of the Johns Hopkins Bloomberg School of Public Health and the Hospital of the University of Elche in Madrid, Spain.

Twelve LATTE-2 trial clinicians were also recruited to obtain the perspective of the providers and their perceptions around prescribing LA ART. Providers were contacted by members of the study team who described study objectives, assessed interest and obtained consent. Dates and times were arranged for these key informant interviews at the local trial clinic or the provider’s office. A flexible guide of open-ended questions was used to direct provider interviews, exploring similar issues to those in patient interviews from a provider’s perspective and how LA ART would be integrated into patient care and clinical management, as well as from an overall health systems perspective.

### Data management and analysis

Patient contact information was managed by LATTE-2 staff who conducted recruitment. The study team used unique identifiers to label interview forms and audiotapes. No identifiers were included in transcriptions of the anonymous interviews.

The data was approached using iterative thematic content analysis [33]. After transcription, interviews were first read multiple times in their entirety. A codebook was developed from an initial coding structure of *a priori* question-based codes from the original field guide and study objectives. Themes emerging from the data were discussed by the two independent coders and added to the codebook during the process of refining the thematic coding structure. All textual data was then coded in Atlas.ti [34] for both *a priori* and emergent domains of interest [35]. Code output was synthesized across these key domains and salient themes were then extracted and developed from that output. Diversity in perceptions, experiences, and views related to LA ART were explored across sampling categories (e.g. provider vs. patient), study sites, and population sub-groups (such as sexual orientation). Based on our interest in understanding and comparing and contrasting experiences of trial participants, the findings reported here include both deductive, question-based themes and themes that emerged from the data. Throughout data collection, saturation of themes was monitored, ensuring that thematic redundancy was established and a point was reached when little new information related to the study domains was emerging.

### Sample characteristics

As seen in Table 1, the sample of interview participants included 11 patients from the U.S. and 16 from Spain. Most participants were male, due to the parent study composition, with 10/11 participants in the U.S. and 15/16 in Spain being men. The mean age was similar across sites, with most participants in their 30s (38 in U.S.; 37 in Spain). Most men were MSM in both locations. The sample was more racially/ethnically diverse in the U.S. (5/11 non-Caucasian) versus Spain (3/16 Latino/South American) reflecting the demographics of the participants of the larger trial. A total of 14 participants across the sites received LA injections every 4 weeks while 13 participants received LA injections every 8 weeks. Twelve key informants (2 per site, with 3 sites in each country) were interviewed including study investigators (3 female and 3 male physicians) and staff (2 female nurses and 4 male study coordinators) from the LATTE-2 sites.

**Table 1:** Select demographic and behavioral characteristics of trial participants (n = 27).

Variables	United States (n = 11)	Spain (n = 16)
Gender	10/11 Male 1/11 Female	15/16 Male 1/16 Female
Age (mean, range)	38 (24–59)	36 (20–51)
Sexual orientation	9/11 MSM 1/11 Heterosexual male 1/11 Heterosexual female	14/16 MSM (1 bisexual) 1/16 Heterosexual male 1/16 Heterosexual female
Race/ethnicity	6/11 Caucasian 2/11 African American 2/11 Asian 1/11 Haitian	13/16 Caucasian/Spanish 3/16 Latino/South American

## Results

### Participant experiences with injections: There are side effects, but they are “worth it”

The majority of trial participants described having some degree of adverse reactions to injections. These generally involved soreness and minor bruising at the injection site for 1–2 days. Some participants reported managing these side effects with non-prescription drugs such as ibuprofen or acetaminophen. A minority of participants described more pronounced reactions, such as hardness at the injection site, fever, and impaired mobility issues in a few cases. Despite these experiences, there was broad agreement that side effects were “worth it,” as seen in the quotes below from trial participants.

*One day is nothing…it’s as if you have a day with a headache*. *You take ibuprofen and that’s it*. *You put up with it*. *It’s temporary*.–Spain, Male trial participant

*It might be painful*, *but it’s better than pills*.–U.S., Male trial participant

Overall, participants relayed the idea that receiving LA ART was simple and easy, and that in general, you “don't have to worry about it (one’s HIV status)” for 1–2 months.

### Comparisons with daily orals: LA ART as convenient and confidential

Participants’ reasons for tolerating adverse effects associated with injections included convenience, greater confidentiality and privacy, and psychosocial or emotional benefits, in comparison with daily oral ART. LA ART was perceived as simple and easy to integrate into one’s daily life, including in the case of travel or non-routine evening or social activities. LA ART was also seen as more “discrete” than pills, with less opportunity for stigma or discrimination or non-desired disclosure of HIV status either through someone seeing pills at one’s house or in a suitcase while traveling, seeing one take pills, or seeing pills delivered to one’s house, etc. Others remarked on the fact that some countries still have laws against PLHIV entering and reported the fear of luggage being searched and immigration officials finding their pills. For some, oral ART was also seen as an unwanted daily reminder of HIV. The quote below brought all of these factors together in one statement from a Spanish trial participant.

*It seems to me that it’s much better because you simply don’t have to worry about anything*. *If you go on a trip*, *you don’t have to bring your pills or take anything at all along*. *You follow your ‘normal life*.*’ You come once a month*. *You get the shot and it’s over*. *You don’t have to be thinking everyday …oh I forgot to take the pill*. *Or …when did I take it last*… *You just don’t worry about anything*. *In reality*, *taking the pill everyday keeps it [HIV] present …and the shot is just once a month…you remember it when you come in and the rest of the time you can basically forget it*.–Spain, Male trial participant

The feeling that LA ART had the potential to reduce internalized stigma related to HIV was also echoed by a U.S. study participant who commented:

*It's less and less stigmatized with the injection*, *because I don't feel like I'm reminding myself of [HIV]…with the injection you go through days and weeks…two months not having to worry about that*, *so it's less stigmatized*.*–*U.S., Male trial participant

There was a significant desire expressed by study participants to feel “normal” again, not feel “sick” all the time and not have to dwell on one’s HIV status. While this sentiment was expressed in both countries, the frequency of its mention was greater in the Spanish context.

*At the beginning I thought…Oh my God…I hope I get over this depression. But, my God…I hope I won’t be taking these pills all my life. Then I went on to the injectable phase…and it was like I saw the light. And I said, God…how easy and convenient this is. It was like seeing the light. And hopefully over time it won’t be every month. Maybe every 2 or 3. That would be even better, right?!? When I was on the pills I just thought ‘I can’t do this and I am sorry but I am done! I am not going to take them, I just don’t want to do this anymore.’ I felt like doing that, but I didn’t*.

*But the injection is a whole other situation*. *It’s just one quick moment and then it’s over*. *You don’t have to deal with it every day …like you do with the pills…*.*and in some ways that just makes you have to continue to think about it each time*.–Spain, Male trial participant

Female participants described LA ART as having the potential to reduce stress and pressure associated with just one more thing they had to worry about, given their multiple roles as mothers, partners, workers and caretakers of other family members.

*I love it because I don't have to take a daily medication*, *so that's just one less thing on my plate that I have to worry about… I definitely feel there's less pressure*. *I like the injection because it's not a daily*, *in my face*, *I have to do this*.*–*U.S., Female trial participant

### Adhering to periodic clinic appointments

Regarding clinic attendance, participants generally reported feeling very comfortable coming to the clinics monthly or bi-monthly to receive their injections (timing was dependent on the study arm they were randomized to) and they expressed feeling well-treated, respected and supported.

However, a few participants expressed concern around the number of clinic visits involved and the potential it created for unwanted HIV status disclosure dynamics as described below. These concerns appeared to emanate from the fact that most participants had disclosed to just a few people in their inner circle of family and friends and that often their employers did not know they were living with HIV. While some (particularly in the U.S.) indicated that stigma had decreased over the years, fears about potential discrimination were still quite salient. In both settings, participants discussed the idea that stigma related to sexual orientation was less than that related to HIV and cited reduced legal restrictions related to gay marriage in both environments.

*I was a little nervous about seeing the doctor so often*. *Even my carpool buddy asked a couple of times*, *‘Wow*. *You go to the doctor a lot*. *They draw a lot of blood*.*’ Then*, *I started saying*, *‘Well*, *I just have an appointment for my roofer*, *and my plumber is going to be coming in a second*.*’ I stopped saying I was going to the doctor so much*.*–*U.S., Male trial participant

The number of visits, however, in this case reflects both study-related visits in addition to injection appointments. In general participants shared that for them, “less is better,” as many participants relayed the hope for quarterly or less frequent injection schedules.

### Appropriate patients and populations for LA ART

Many trial participants felt that LA ART could be appropriate for “everyone” living with HIV, and did not immediately distinguish sub-groups that would be particularly appropriate candidates. However, upon further probing, participants shared that particularly “good candidates” for LA ART would involve those who are “tolerant of needles,” and perhaps younger people or those who do not take other medications, those with “hectic” or active lifestyles, or what were termed by some (both patients and providers) as “unstable” populations including people who might be homeless, substance users or the mentally ill or disabled.

*I realized how lucky I am*. *I have a stable home*. *I have family support*. *I have income*. *I have a job*. *I have stability*. *There's so many people that don't have that*, *that are living on the streets or they're in shelters or they don't have a consistent place to stay*. *I know through what the clinic offers*, *people can come in and see all their doctors in one spot*, *things like that to help make things convenient for them or they can get their meal there if they want*. *So they have some sort of [support]—and that's something that somebody who doesn't have stability—if you don't know where you're going every night—to make sure that you take your medication every night*. *If you can know that once a month or every other month you take an injection*, *then that's one less worry*.*–*U.S., Female trial participant

There was only one participant (an older gay man) in the U.S. who indicated that he may be fine sticking with daily oral pills because he is taking various other medications anyway.

*I’m used to taking three pills a day and I’m very good at it*. *I’m a little compulsive anyway so I think I could go either direction*, *but I think people that are not used to taking anything*, *and there a lot of people like that*, *would prefer this (LA ART)*.*–*U.S., Male trial participant

However, most PLHIV interviewed indicated they would recommend LA ART to anyone living with HIV and several commented their “friends are so jealous” among those who knew that they were able to receive LA ART through the trial. One participant relayed: “90% of the people will prefer this method,” underscoring the almost unanimous sentiment around this delivery method.

### Provider views regarding the clinical management of LA ART

Providers were also generally supportive of LA ART as an important option for PLHIV but were not as definitively positive as patients. Providers commented on the need to consider and decide on a given ART regimen on a case-by-case basis and believed that many patients “can take pills just fine.” They also highlighted that patients would still need to be able to come to the clinic as scheduled, and in turn LA ART did not completely eliminate the need for adherence or related barriers. Additionally, they relayed some concerns about resistance and the more complex clinical management of LA compared to oral ART, given that the drug would stay in the body for a longer period of time in the case there was a need to take a given patient off the regimen.

*My concern with injections is this*: *when you have someone who's just not compliant*. *If they're not compliant and they miss two or three oral doses*, *it's not the end of the world*. *If you're not compliant with every eight weeks*, *that could be an issue*. *So you've got to get people who understand the importance of adhering*. *Now*, *we both know you've got lots of leeway with these drugs*. *It's not like*, *"You're two days late*. *Oh my god*, *you're going to be resistant*.*" But those are the patients*.–U.S., Study site nurse

*For many patients*, *yes…the profile must be well perceived because it is true that poor adhering patients can be very good candidates*, *since you inject the medication and you know…it’s there…It’s similar but very different because here we have a medication injected*, *and the fear is that the patient does not reappear*. *Then the medication is at descending levels over months*, *and therefore choosing a selective perception with the virus*. *So yes*, *this concerns us somehow…It is true that the tolerance is handled very well by the fact that it is oral phase and then injection phase*, *but after…the injection of the medication and it cannot be removed*. *There it is administered and if there is an allergy or intolerance*…*it is true that the protocol I say that is fine*, *because there is a margin of five months to really assess if there is an intolerance to oral medication*, *which is equivalent to the injection but I guess an exception could exist with the patient*, *the fact that the medication cannot be removed once it is given*… *it may be many months without really knowing its secondary effects*.–Spain, Study site physician

### Perspectives on implementation and delivery of LA ART

Participants generally agreed on the need for “skilled” or “trained” professionals to administer injections such as a doctor or nurse. In turn, the majority of participants indicated that LA ART is best suited to ongoing clinical care, although some participants mentioned the idea of receiving injections at other sites such as community health centers or pharmacies in the U.S. There was some disagreement regarding whether primary versus HIV-specific care would be the appropriate venue.

The fact that Spain has a single payer (government) versus multi-payer health care system was also discussed in relation to how to best manage patient flow and care with LA ART. Given the larger HIV clinics in the trial in Spain, providers worried about patient flow and where and how to give injections. In the U.S., on the other hand, with fewer patients in the clinics, providers mentioned concerns about being able to keep stocks with small numbers of patients in clinics. In the U.S., participants were asked about willingness to pay for LA ART and on average people indicated that they would be willing to pay approximately $184 ($50-$500) per month, most likely as a co-pay to their private or state-run insurance coverage, with a median of $100/month.

## Discussion

Study participants perceived LA ART as a highly acceptable and desirable treatment option. The convenience and peace of mind of a monthly or semi-monthly injection versus daily pills was described by participants as having significant potential for improved adherence and therefore improved HIV treatment outcomes. Study participants varied in age and other demographics, however we observed consistency in results regarding satisfaction with injectable ART across groups. Just as the effect of a reduced number of daily pills on adherence and quality of life in HIV-infected patients was documented with the advent of single-tablet regimens (STRs) [36–38], LA ART may move patients closer to achieving long-term treatment success.

While providers were also generally supportive of LA ART as a treatment option, they also expressed some concerns regarding practical considerations for roll-out. While some patients believed that “everyone” would benefit from LA ART, providers were more nuanced regarding the most appropriate patients for this delivery method. Providers felt that they had an important responsibility in ensuring that patients were adherent with their monthly or bi-monthly dosing regimen. Findings suggest the need to support patient-provider communication around evolving therapeutic options including the need for guidelines and screening and assessment tools to decide who are the best candidates for LA ART if it is proven effective, and when and how to transition patients between regimens based on changing social and clinical dynamics.

The experiences and perspectives of participants in this study around disclosure dynamics, stigma, adhering to clinic visits, and medication side effects align with some of the factors that have been well documented in the literature on predictors of ART adherence [16, 39]. As expressed by participants, LA ART helps address some of these barriers while others will require examination specifically in the context of LA ART and would need to be addressed in “real world” roll-out. For example, LA ART was perceived by many participants as a means to reduce HIV stigma. At the same time, this finding highlights the continued salience of stigma in their lives and the potentially negative role it can play in treatment adherence [25, 40, 41]. While LA ART is a promising biomedical tool and possible stigma reduction strategy, comprehensively addressing structural HIV stigma remains critical. These findings underscore the need to integrate structural and biomedical interventions to improve HIV outcomes [42].

Findings also have implications for future research including the need to quantitatively assess factors potentially related to LA ART acceptability among PLHIV such as patient-provider communication around LA ART, HIV and ART-related stigma (internalized and experienced), socio-economic status and stability, gender and care taking roles and burden, prior injection use experiences (injectable contraception, injectable chronic medicines, illicit injection drug use) and HIV disclosure within social and professional networks. Additionally, future efforts should qualitatively explore longitudinal experiences with LA ART among a more diverse group of PLHIV and providers, including diversity in terms of gender, race/ethnicity, socio-economic stability and geographic context (U.S., Europe, Latin America and Africa) and examine changes in views, experiences and preferences for LA versus oral ART over time.

This study has several limitations. First, the study was cross-sectional in nature and did not allow us to capture variations in experiences with LA ART over time. Second, the study relied on a sample of “motivated” participants from a clinical trial, all of whom had achieved viral suppression in the oral ART lead-in phase of the trial. These limitations of the sample may have influenced the generally positive experience of participants with long-acting treatment. However, findings reveal strong and consistent support for LA ART across the study settings as a potentially paradigm-changing treatment tool to address both practical and emotional barriers to daily oral ART adherence.
